# Comparative Transcriptome Analysis Reveals the Protective Mechanism of Glycyrrhinic Acid for Deoxynivalenol-Induced Inflammation and Apoptosis in IPEC-J2 Cells

**DOI:** 10.1155/2020/5974157

**Published:** 2020-10-24

**Authors:** Xiaoxiang Xu, Guorong Yan, Juan Chang, Ping Wang, Qingqiang Yin, Chaoqi Liu, Qun Zhu, Fushan Lu

**Affiliations:** ^1^College of Animal Science and Veterinary Medicine, Henan Agricultural University, Zhengzhou 450002, China; ^2^Institute of Photomedicine, Shanghai Skin Disease Hospital, Tongji University School of Medicine, Shanghai 200443, China; ^3^Henan Delin Biological Product Co. Ltd., Xinxiang 453000, China; ^4^Henan Puai Feed Co. Ltd., Zhoukou 466000, China

## Abstract

Deoxynivalenol (DON) is the most common mycotoxin that frequently contaminates human food and animal feed, resulting in intestinal diseases and systemic immunosuppression. Glycyrrhinic acid (GA) exhibits various pharmacological activities. To investigate the protective mechanism of GA for DON-induced inflammation and apoptosis in IPEC-J2 cells, RNA-seq analysis was used in the current study. The IPEC-J2 cells were treated with the control group (CON), 0.5 *μ*g/mL DON, 400 *μ*g/mL GA, and 400 *μ*g/mL GA+0.5 *μ*g/mL DON (GAD) for 6 h. Results showed that 0.5 *μ*g/mL DON exposure for 6 h could induce oxidative stress, inflammation, and apoptosis in IPEC-J2 cells. GA addition could specifically promote the proliferation of DON-induced IPEC-J2 cells in a dose- and time-dependent manner. In addition, GA addition significantly increased Bcl-2 gene expression (*P* < 0.05) and superoxide dismutase and catalase activities (*P* < 0.01) and decreased lactate dehydrogenase release, the contents of malonaldehyde, IL-8, and NF-*κ*B (*P* < 0.05), the relative mRNA abundances of IL-6, IL-8, TNF-*α*, COX-2, NF-*κ*B, Bax, and caspase 3 (*P* < 0.01), and the protein expressions of Bax and TNF-*α*. Moreover, a total of 1576, 289, 1398, and 154 differentially expressed genes were identified in CON vs. DON, CON vs. GA, CON vs. GAD, and DON vs. GAD, respectively. Transcriptome analysis revealed that MAPK, TNF, and NF-*κ*B signaling pathways and some chemokines played significant roles in the regulation of inflammation and apoptosis induced by DON. GA may alleviate DON cytotoxicity via the TNF signaling pathway by downregulating IL-15, CCL5, and other gene expressions. These results indicated that GA could alleviate DON-induced oxidative stress, inflammation, and apoptosis via the TNF signaling pathway in IPEC-J2 cells.

## 1. Introduction

The gastrointestinal tract of animals plays an important role in the digestion and absorption of various nutrients and provides a pivotal barrier against food contaminants and other harmful irritants such as toxins, stress, and pathogens [1, 2]. The injury gastrointestinal tract will cause barrier dysfunction, leading to inflammatory bowel diseases, necrotizing enterocolitis, and other intestinal diseases [3]. Deoxynivalenol (DON), called colloquially vomitoxin, is one of the most common mycotoxins mainly produced by *Fusarium graminearum* and *Fusarium culmorum* to contaminate human food and animal feed [4, 5]. In general, the primary target of DON is the intestinal tract. Consumption of DON-contaminated food or feed brings about the compromised intestinal epithelial barrier function [6], the augment of intestinal permeability [7], disordered intestinal structure [8], and lower nutrient absorption [9], which leads to intestinal diseases and systemic immunosuppression. In addition, swine is the most sensitive domestic animal to DON. The IPEC-J2 cell line, isolated from the jejunal epithelium of the neonatal piglet, was widely used as an *in vitro* model for studying intestinal functions [10].

According to previous studies, DON exposure could induce cytotoxicity, oxidative stress, inflammation, and apoptosis and influence cell growth and functions [11–13]. DON triggers epithelial inflammation and oxidative stress by stimulating proinflammatory cytokine production in different epithelial cells as well as activates prototypical signaling pathways linked to immunity and inflammation such as the nuclear factor- (NF-) *κ*B and mitogen-activated protein kinase (MAPK) [14, 15]. The MAPK pathway plays an important role in regulating cell proliferation, inflammation, and immune reactions in the intestine, which consists of extracellular signal-regulated kinase (ERK), p38, and stress-activated protein kinase (JNK) pathways [16]. NF-*κ*B is essential for the immune system, which can modulate cytokine expression and effector enzymes, as well as bind to various receptors involved in immunization [17]. External factors such as toxins and pathogens could stimulate the activation of the MAPK and NF-*κ*B pathways to cause cell apoptosis [18]. Many researches have uncovered the toxic effects of DON on intestinal cells; however, the molecular mechanism underlying DON-induced inflammation and apoptosis of intestinal epithelial cells is not completely clear. Therefore, it is very imperative to fully reveal the toxic effects of DON on intestinal cells and to find effective nutritional and protective strategies for alleviating DON-induced intestinal damage to maintain animal health.

Traditional Chinese medicines have been recognized as good feed additives in improving animal production performance, immunity, and disease resistance [19, 20]. Licorice, a perennial herb, has well-known detoxifying effects [21]. Glycyrrhinic acid (GA) is a botanical extract in licorice with various pharmacological activities including anti-inflammatory, immune regulation, antioxidation, antivirus, anticancer, and hypolipidemic effects [22–24]. Licorice extracts have been reported for clinical use in the treatment of liver injury [25] as well as for preventing diseases and promoting animal growth and meat quality [26]. Although many studies have confirmed the anti-inflammatory and liver protection effects of GA, the intestinal protective effect of GA against DON-induced toxicity, inflammation, and apoptosis has not been found.

To further compare the differential gene expression profiles and elucidate the repairing mechanisms of GA against inflammation and apoptosis in DON-stimulated IPEC-J2 cells, the high-throughput RNA sequencing (RNA-seq) was used in this study. The results will contribute to the identification of functional genes to provide a more comprehensive understanding of GA for suppressing inflammation and apoptosis in DON-stimulated IPEC-J2 cells. It will lay a theoretical basis for the application of GA as a feed additive for alleviating DON cytotoxicity to improve animal health and production performance.

## 2. Materials and Methods

### 2.1. Materials and Reagents

Glycyrrhinic acid was obtained from Luoyang Lansealy Technology Co., Ltd. (Luoyang, Henan, China). DON was purchased from Sigma-Aldrich (St. Louis, MO, USA), dissolved in dimethylsulfoxide (DMSO) (Beijing Solarbio Biotechnology Co., Ltd., Beijing, China), formulated into 1 mg/mL stock solution, and stored at -20°C. Phosphate-buffered saline (PBS), penicillin- (10000 U/mL) streptomycin (10 mg/mL), 0.25% pancreatin with or without ethylenediaminetetraacetic acid (EDTA), and 3-(4,5-dimethylthiazol-2-yl)-2,5-diphenyltetrazolium bromide (MTT) were purchased from Beijing Solarbio Biotechnology Co., Ltd., Beijing, China. High-glucose Dulbecco's Modified Eagle's Medium (H-G DMEM) and fetal bovine serum (FBS) were purchased from Biological Industries (Kibbutz Beit-Haemek, Israel). The Annexin V-FITC/PI kit was purchased from Shanghai Qihai Futai Biotechnology Co., Ltd., Shanghai, China. The IL-8, NF-*κ*B, and caspase 3 concentrations assay kits were purchased from Jiangsu Meimian Industrial Co., Ltd., Jiangsu, China. Trizol reagent was purchased from Invitrogen (Grand Island, NY, USA). The reverse transcription kit and TB GREEN kit were purchased from TaKaRa Bio Inc. (Dalian, China). All cell culture materials were purchased from Corning Costar (Cambridge, NY, USA). Rabbit polyclonal antibody of Bax (abs119724) and TNF-*α* (abs123966) and goat anti-rabbit antibody of IgG were purchased from Absin Bioscience Inc., Shanghai, China. The *β*-actin was purchased from Bioworld Technology Inc., Nanjing, China.

### 2.2. Cell Culture and Treatments

The IPEC-J2 cell line was obtained from the College of Animal Science and Technology, Jiangxi Agricultural University (Jiangxi, China) and cultured in H-G DMEM supplemented with 10% FBS, 100 U/mL penicillin, and 100 *μ*g/mL streptomycin at 37°C in an incubator with 5% CO_2_. When the confluence of cells reached 80-90%, cells were seeded into 96-well or 6-well plates and cultured for 24 h prior to different treatments. Based on the previous research in our laboratory (Xu et al., 2020), 0.5 *μ*g/mL DON treated for 6 h was selected to make the cell damage model. GA and DON were diluted with H-G DMEM without serum and antibiotics.

### 2.3. Cell Viability

The effect of GA on alleviating DON cytotoxicity was determined using MTT assay. The cells (1 × 10^4^ cells/well) were seeded in 96-well plates and cultured for 24 h and subsequently incubated with an increasing concentration of GA (0, 50, 100, 200, 400, and 800 *μ*g/mL) for 3, 6, 12, and 24 h, respectively. After the optimal GA concentration was selected, the cells were incubated with 0.5 *μ*g/mL DON and the optimal GA concentration for 6 h. Then, each well was added with 10 *μ*L 5 mg/mL MTT and incubated for 4 h at 37°C. Thereafter, the supernatant was removed and 150 *μ*L DMSO was added to each well to solubilize the formazan. The plate was shaken at room temperature for 10 min. The absorbance was measured at 490 nm wavelength with a reference wavelength of 630 nm by an ELx800 microplate reader (BioTek Instruments Inc., Winooski, VT, USA).

### 2.4. Annexin V-FITC/PI Apoptosis Assay

The apoptosis cells were examined using the Annexin V-FITC apoptosis detection kit. The cells (5 × 10^5^ cells/well) were seeded in a 6-well plate and incubated for 24 h and then subsequently treated with the control group (CON), 0.5 *μ*g/mL DON, 400 *μ*g/mL GA, and 400 *μ*g/mL GA+0.5 *μ*g/mL DON (GAD) for 6 h. After four treatments, the cells were digested by pancreatin without EDTA and centrifuged at 1000 rpm for 5 min to remove the medium. Then, the precipitation was washed twice with PBS and resuspended in 100 *μ*L 1x binding buffer and stained with 5 *μ*L Annexin V-FITC and 5 *μ*L PI at room temperature in the dark for 15 min. Finally, 400 *μ*L 1x binding buffer was added to each tube, and cell apoptosis levels were analyzed by flow cytometry (Em 530 nm, Ex 488 nm, Becton Dickinson Company, NJ, USA).

### 2.5. Determinations of IL-8, NF-*κ*B, and Caspase 3

IPEC-J2 cells (5 × 10^5^ cells/well) were seeded in 6-well plates and cultured for 24 h. Following four treatments (control, 0.5 *μ*g/mL DON, 400 *μ*g/mL GA, and 400 *μ*g/mL GA+0.5 *μ*g/mL DON) for 6 h, the cell supernatants were collected to determine IL-8, NF-*κ*B, and caspase 3 contents, and the cells were washed twice with PBS to extract the proteins for further use. The IL-8, NF-*κ*B, and caspase 3 concentration assays were quantified by enzyme-linked immunosorbent assay (ELISA) in accordance with the manufacturer's instructions.

### 2.6. RNA Extraction, Library Construction, and Sequencing

IPEC-J2 cells (5 × 10^5^ cells/well) were seeded in 6-well plates and cultured for 24 h. Following four treatments (control, 0.5 *μ*g/mL DON, 400 *μ*g/mL GA, and 400 *μ*g/mL GA+0.5 *μ*g/mL DON) for 6 h, the cells were washed twice with PBS and then harvested. Total RNA was isolated using Trizol (Invitrogen Corporation, Grand Island, NY, USA) according to the manufacturer's instructions and stored at -80°C. The RNA quantity and purity were assessed on an Agilent 2100 Bioanalyzer (Agilent Technologies, Santa Clara, CA, USA), and the RNA integrity was checked by agarose gel electrophoresis. The purified mRNA was enriched by Oligo(dT) beads and fragmented into 300 bp by Mg^2+^ ion fragmentation buffer. Then, the fragmented mRNA was reversely transcripted into cDNA with 6 bp random primers. After that, the second-strand cDNA was synthesized using the first-strand cDNA as a template. The synthesized product was purified with a PCR purification kit (Qiagen, Venlo, The Netherlands), followed by end repair, poly(A) addition, and Illumina sequencing adapter ligation. The sequencing libraries were made, and the Agilent 2100 Bioanalyzer was used to check their sizes. Finally, sequencing libraries were sequenced using the Illumina HiSeq 4000 platform (Illumina, San Diego, CA, USA) with a paired-end (PE 150 bp) sequencing strategy.

### 2.7. Data Analysis of RNA Sequencing

The quality of raw reads was evaluated and cleaned by trimming the 3′ adapter sequence using cutadapt [27]. The raw reads with an average sequencing accuracy below 99.9% were removed. The clean reads were aligned to the reference porcine genome assembly Sus scrofa 11.1 using HISAT2 with default parameters [28]. The mapping results of different genomic regions were evaluated. Read counts for each gene were calculated using HTSeq with union strategy [29]. In order to avoid the effects of sequencing coverage and gene length, read counts were normalized into fragments per kilobase of exon model per million mapped fragments (FPKM), which were used as input for the following analysis. Pearson correlation coefficient was calculated between different samples using the expression values of all detected genes. Moreover, principal component (PC) analysis was also performed among all samples using all detected genes. Differential expression analysis was performed on DESeq2 (Bioconductor version 1.6.2), and genes with the threshold of *P* < 0.05 and the absolute value of log2 (fold change) > 1 among two groups were regarded as the differentially expressed genes (DEGs).

### 2.8. GO Functional Annotation and KEGG Pathway Enrichment Analyses

In order to deeply gain insights into the functions of DEGs obtained from different treatments, Gene Ontology (GO) annotation and Kyoto Encyclopedia of Genes and Genomes (KEGG) pathway enrichment analyses were carried out. GO annotation including biological process (BP), molecular function (MF), and cellular component (CC) was performed using the topGO package. The significance of genes annotated in a GO term was calculated by hypergeometric distribution. The enrichment of genes involved in the same biological functions facilitated the understanding of gene biological functions. KEGG was a pathway-related database for understanding high-level functions and utilities of the biological systems, and a cluster profile package was used for this enrichment.

### 2.9. PPI Network Construction and Module Analyses

To establish protein-protein interaction (PPI) network of interested DEGs, the Search Tool for the Retrieval of Interacting Genes/Proteins (STRING, version 11.0, https://string-db.org/) database was used, which covered interactive relationships of proteins obtained from experiments, data mining, and homological prediction for various species [30]. The minimum required interaction score was set as high (0.90) for two different DEGs. Then, the Cytoscape tools (v3.6.1, http://cytoscape.org/) was used for the visualization of PPI networks. For seeking hub genes and significant network, the cytoHubba and Molecular Complex Deletion (MCODE) plug-ins for Cytoscape were downloaded to determine hub genes using the Maximal Clique Centrality (MCC) ranking method and significant network modules with default parameters.

### 2.10. Real-Time Quantitative PCR

Four treatments and the RNA extraction were carried out as above. About 1 *μ*g total RNA was used to erase gDNA at 42°C for 2 min and then reversely transcripted into cDNA using the TB GREEN kit (TaKaRa Bio Inc., Dalian, China). Real-time PCR was performed with the CFX Connect™ Real-Time PCR Detection System (Bio-Rad, Hercules, CA, USA) in a 20 *μ*L PCR reaction system containing 2 *μ*L cDNA, 1 *μ*L reverse primer,1 *μ*L forward primer, 10 *μ*L SYBR mixture, and 6 *μ*L deionized water. The GAPDH was used as a housekeeping gene to normalize the gene levels, and the RT-PCR data were analyzed using the 2^-*ΔΔ*CT^ method (Livak and Schmittgen, 2001). All the primers used in this study were synthesized by Shanghai Shenggong Biotech Co., Ltd., Shanghai, China (Supplementary Table 1).

### 2.11. Western Blot Analysis

After four treatments, total proteins of the cells were extracted with RIPA buffer (EpiZyme Biotechnology, Shanghai, China), and concentrations were determined by the BCA protein assay kit (EpiZyme Biotechnology, Shanghai, China). The equal amounts of proteins were separated by sodium dodecyl sulfate-polyacrylamide gel electrophoresis (SDS-PAGE) and transferred onto the polyvinylidene difluoride (PVDF) membranes. The membranes were blocked with TBST buffer containing 5% skim milk powder for 2 h, followed by incubation with the primary antibodies at 4°C overnight, and then further incubated with the secondary antibodies for 2 h at room temperature. The immunopositive bands were visualized by enhanced chemiluminescence.

### 2.12. Statistical Analysis

The data were expressed as means ± SD. There were 3 replications for each treatment. Statistical analysis was analyzed using the one-way ANOVA method (Duncan's multiple comparison test) with the SPSS 20.0 software (Sishu Software, Shanghai Co., Ltd., Shanghai, China). All graphs were generated using GraphPad Prism 8. Statistical differences were considered significant at *P* < 0.05.

## 3. Results

### 3.1. GA Alleviated Cytotoxicity and Oxidative Stress in the DON-Induced IPEC-J2 Cells

**Figure 1:**
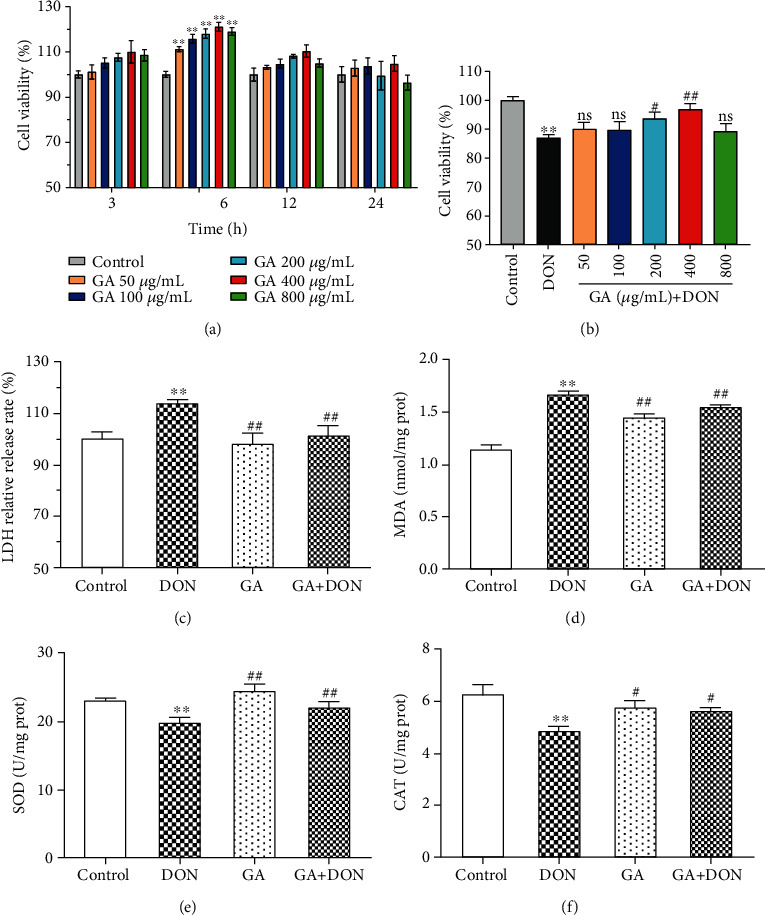
Cell viability and status affected by GA and DON. (a) Effects of different GA concentrations on cell viability at different incubation times (*n* = 6). (b) Effects of 0.5 *μ*g/mL DON and different GA concentrations on cell viability for 6 h incubation (*n* = 6). (c–f) Effects of GA on LDH release and antioxidant indexes in DON-induced cells (*n* = 3). DON: the single DON (0.5 *μ*g/mL) group; GA: the single GA (400 *μ*g/mL) group; GA+DON: 400 *μ*g/mL GA+0.5 *μ*g/mL DON group. ^∗∗^*P* < 0.01 indicates the significant difference, compared with the control group; ^#^*P* < 0.05 and ^##^*P* < 0.01 indicate the significant difference, compared with the DON group; “ns” indicates the insignificant difference, compared with the DON group.

To study the effects of GA on IPEC-J2 cells, the cell viability evaluation was performed using MTT assay.  Figure 1(a) shows that GA promoted cell proliferation in a dose- and time-dependent manner, and cell viabilities were significantly increased when cells were treated with GA for 6 h (*P* < 0.01). Therefore, 6 h was set as the optimal reaction time to confirm the effects of GA on alleviating DON-induced cells. As shown in Figure 1(b), the treatment with 0.5 *μ*g/mL DON for 6 h significantly decreased cell viability (*P* < 0.01); however, 400 *μ*g/mL GA addition could markedly enhance cell viability induced by DON exposure (*P* < 0.01). Therefore, experimental condition with 400 *μ*g/mL GA and 6 h reaction time was employed in the subsequent experiments.

The results in Figures 1(c)–1(f) showed that DON at a concentration of 0.5 *μ*g/mL for 6 h significantly increased LDH release and malonaldehyde (MDA) levels and decreased superoxide dismutase (SOD) and catalase (CAT) activities, compared with the control group (*P* < 0.01). However, GA addition significantly decreased LDH release and MDA level (*P* < 0.01) and increased the activities of SOD (*P* < 0.01) and CAT (*P* < 0.05). The above results suggested that DON exposure could cause serious cell damage, and GA could efficiently alleviate the cytotoxicity and oxidative stress in DON-induced cells.

### 3.2. GA Alleviated IPEC-J2 Cell Apoptosis and Inflammation Induced by DON

**Figure 2:**
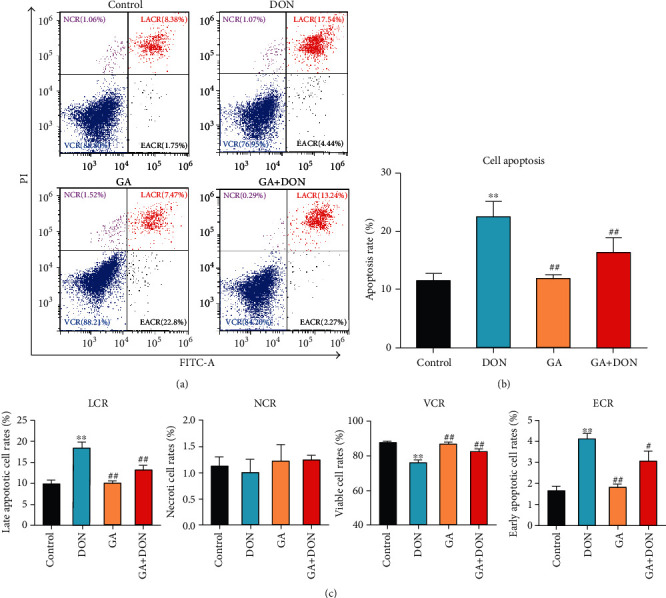
GA alleviating cytotoxicity of DON-induced IPEC-J2 cells. (a) Flow cytometry analysis of Annexin V/FITC/PI staining cells; LACR, NCR, VCR, and EACR represent the late apoptotic cell rates, necrotic cell rates, viable cell rates, and early apoptotic cell rates, respectively. (b) Quantification of the total cell apoptotic rates (early apoptotic cell rates+late apoptotic cell rates). (c) Quantification and analysis of the late apoptotic cell rates, necrotic cell rates, viable cell rates, and early apoptotic cell rates in four groups. DON: the single DON (0.5 *μ*g/mL) group; GA: the single GA (400 *μ*g/mL) group; GA+DON: 400 *μ*g/mL GA+0.5 *μ*g/mL DON group. ^∗∗^*P* < 0.01 indicates the significant difference, compared with the control group; ^#^*P* < 0.05 and ^##^*P* < 0.01 indicate the significant difference, compared with the DON group.

The results of Annexin V/FITC/PI staining in Figures 2(a) and 2(b) showed that the total apoptotic rate of IPEC-J2 cells induced by DON was significantly increased compared with the control group (*P* < 0.01), whereas GA addition significantly decreased it (*P* < 0.01).  Figure 2(c) shows that DON significantly decreased the viable cell rates and increased the late and early apoptotic cell rates (*P* < 0.01); however, GA addition significantly increased the viable cell rates (*P* < 0.01) and decreased the late apoptotic cell rates (*P* < 0.01) and early apoptotic cell rates (*P* < 0.05).

**Figure 3:**
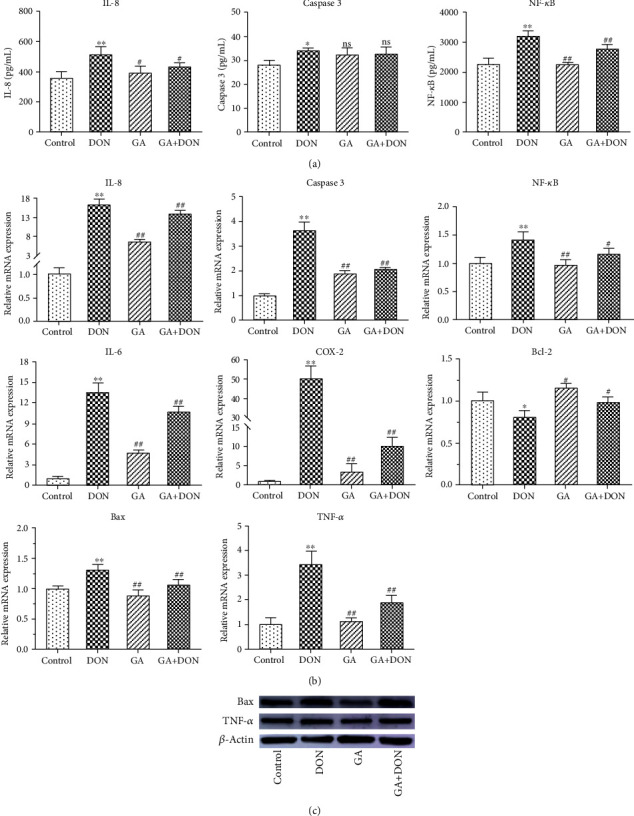
GA alleviating IPEC-J2 cell apoptosis and inflammation induced by DON. (a) The contents of IL-8, caspase 3, and NF-*κ*B. (b) Effects of GA on regulating the relative mRNA abundances of apoptotic and inflammatory genes in DON-induced IPEC-J2 cells. (c) The relative protein expressions of Bax and TNF-*α* in four groups. DON: the single DON (0.5 *μ*g/mL) group; GA: the single GA (400 *μ*g/mL) group; GA+DON: 400 *μ*g/mL GA+0.5 *μ*g/mL DON group. ^∗^*P* < 0.05 and ^∗∗^*P* < 0.01 indicate the significant difference, compared with the control group; ^#^*P* < 0.05 and ^##^*P* < 0.01 indicate the significant difference, “ns” indicates *P* > 0.05, compared with the DON group.

As shown in Figures 3(a) and 3(b), DON exposure significantly increased the contents and the relative mRNA abundances of IL-8, caspase 3, and NF-*κ*B (*P* < 0.05), upregulated the relative mRNA abundances of Bax, IL-6, TNF-*α*, and COX-2 (*P* < 0.01), and downregulated the Bcl-2 mRNA abundances (*P* < 0.05). However, compared with the DON group, GA addition significantly decreased the contents of IL-8 (*P* < 0.05) and NF-*κ*B (*P* < 0.01), downregulated the mRNA abundances of IL-8, NF-*κ*B, Bax, IL-6, TNF-*α*, and COX-2 (*P* < 0.01), and upregulated the Bcl-2 mRNA abundance (*P* < 0.05).  Figure 3(c) shows that the protein expressions of Bax and TNF-*α* were increased by DON exposure but decreased by GA addition, corresponding with the changes of mRNA abundances. It was summarized that GA addition could alleviate apoptosis and inflammation in DON-induced IPEC-J2 cells.

### 3.3. RNA-seq Data Analyses

**Figure 4:**
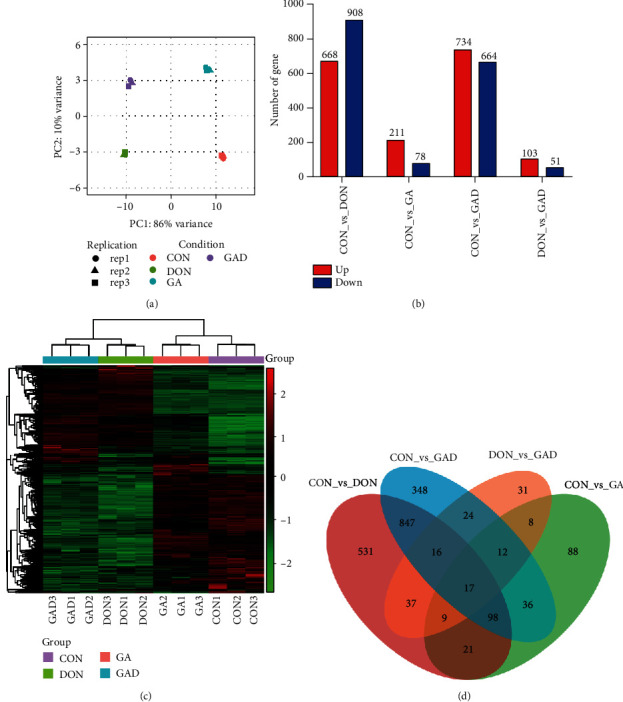
Differentially expressed genes (DEGs) profiling by RNA-seq analysis. (a) Principal component analysis of 12 sample relationships. (b) The DEG number of upregulated and downregulated genes in the comparison between different treatment groups. (c) Hierarchical clustering and heat map of DEGs. (d) Venn diagrams of DEGs. CON: control group; DON: the single DON (0.5 *μ*g/mL) group; GA: the single GA (400 *μ*g/mL) group; GAD: 400 *μ*g/mL GA+0.5 *μ*g/mL DON group.

In order to deeply uncover the molecular mechanism of GA for alleviating IPEC-J2 cell apoptosis and inflammation induced by DON, RNA-seq was performed in four groups including CON, DON, GA, and GAD. The sequencing quality of Q20 (base error < 1%) and Q30 (base error < 0.1%) was 97.96% and 94.51%, respectively. After removing reads containing poly-N and adapters and filtering low-quality reads, approximately 42 million clean reads (93.59%) were kept, in which the reads with an average of 96.64% were mapped to the reference genome (Supplementary Table 2). The correlation plot showed distinct groups for different samples using unsupervised hierarchical clustering. After PCA analysis, the first two PC were used for evaluating the relationship of 12 samples according to their gene expression profiles. PC1 and PC2 explained 86% and 10% of the total variations, respectively, and the samples in different groups were gathered tightly (Figure 4(a)). These results showed a satisfactory data quality of RNA-seq, and three biological replications in each treatment displayed a homogeneity regarding whole gene expressions.

An expression matrix including 21278 genes from RNA-seq was obtained. A total of 1576 DEGs (668 upregulated and 908 downregulated), 289 DEGs (211 upregulated and 78 downregulated), and 1398 DEGs (734 upregulated and 664 downregulated) were identified in the DON, GA, and GAD groups, compared with the control group, respectively.  Figure 4(b) shows the upregulated and downregulated genes in CON vs. DON, CON vs. GA, CON vs. GAD, and DON vs. GAD, respectively. Subsequently, the DEG clusters were analyzed in a heat map, which showed a more similarity among samples from the same treatment based on those genes (Figure 4(c)). Three biological replications in each treatment were clustered together with almost identical distance using DEGs by hierarchical clustering, indicating good reproducibility of each treatment. In addition, the coexpressed DEGs in DON vs. CON, GA vs. CON, GAD vs. CON, and GAD vs. DON were analyzed. As shown in the Venn diagram (Figure 4(d)), there were 79 coexpressed DEGs in CON vs. DON and DON vs. GAD. Interestingly, there were 37 downregulated DEGs in CON vs. DON, but they were upregulated in DON vs. GAD; there were 26 upregulated DEGs in CON vs. DON, but they were downregulated in DON vs. GAD (Table 1).

### 3.4. GO Annotation and KEGG Enrichment

**Figure 5:**
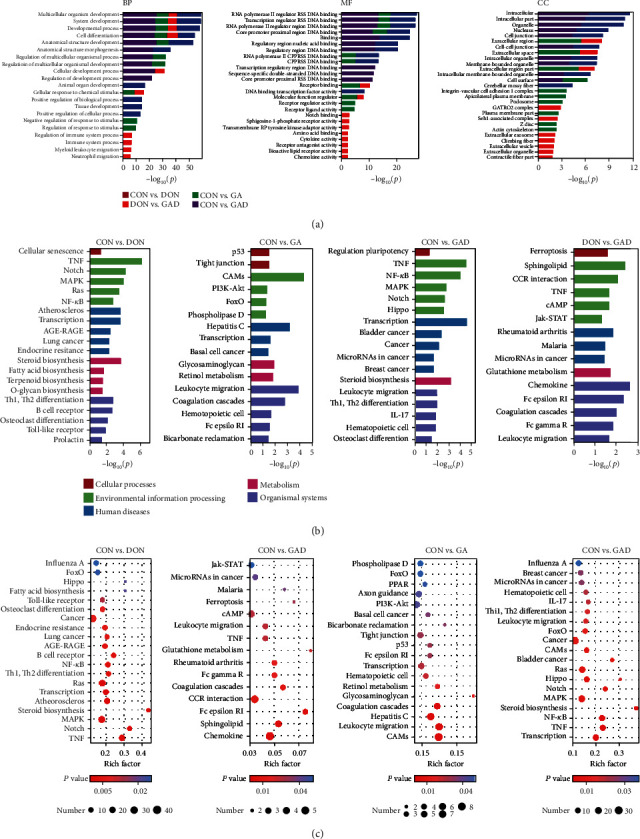
Functional annotation and pathway enrichment results. (a) Gene Ontology (GO) annotation of DEGs in four different comparisons. The top 10 functional classified GO terms of DEGs annotated by the subontology of GO analysis including biological processes (BP), molecular function (MF), and cellular components (CC). (b, c) Kyoto Encyclopedia of Genes and Genomes (KEGG) pathway enrichment of DEGs for four different comparisons. (b) Top 10 classifications regarding different KEGG pathways. (c) Scatterplot of top 20 pathways in KEGG enrichment.

To understand the biological processes and pathway of DEGs, they were functionally annotated and enriched into GO and KEGG databases, respectively. GO analysis were performed regarding three main ontologies: biological process (BP), molecular function (MF), and cellular component (CC). The top 10 GO terms for DEGs identified from CON vs. DON, CON vs. GA, CON vs. GAD, and DON vs. GAD are summarized in Figure 5(a). In the BP level, these four clusters of DEGs were mainly annotated as system development and cell differentiation in the top 10 terms; however, some DEGs from CON vs. GA were annotated as a cellular response to a stimulus, and some DEGs from CON vs. GAD were annotated as the immune system process. In the MF level, almost all the DEGs were annotated as sequence-specific DNA binding except for DON vs. GAD. In the CC level, DEGs were intensively annotated as intracellular organelle in CON vs. DON and CON vs. GAD; however, almost all the DEGs were annotated as an extracellular region or cell membrane in CON vs. GA, which showed a similar annotation as DON vs. GAD to some extent.

KEGG pathway enrichments were conducted for DEGs in four different comparisons mentioned above to identify pathways that changed significantly under different experimental conditions. All the DEGs were enriched according to cellular processes, environmental information processing, human diseases, metabolism, and organismal systems (Figures 5(b) and 5(c)). The DEGs in the CON vs. DON group were mainly involved in the following three pathways: (1) many signal transduction pathways including TNF, Notch, MAPK, Ras, and NF-*κ*B; (2) some immune-related signal pathways including Th1 and Th2 cell differentiation, B cell receptor, and Toll-like receptor; (3) three metabolic pathways including steroid biosynthesis, biosynthesis of unsaturated fatty acids, and terpenoid backbone biosynthesis (Supplementary Table 3). In CON vs. GA, the signal pathways included cell adhesion molecules (CAMs), leukocyte transendothelial migration, complement and coagulation cascades, and PI3K-Akt (Supplementary Table 4). There were more than 900 DEGs to be shared between CON vs. DON and CON vs. GAD; their enriched pathways were similar, which was related to signal transduction and immune systems (Supplementary Table 5). Interestingly, some same pathways such as TNF, cAMP, and leukocyte transendothelial migration were identified in DON vs. GAD and CON vs. DON, in which the Jak-STAT signaling pathway was unique. In addition, immune systems including the chemokine signaling pathway, Fc epsilon RI signaling pathway, complement and coagulation cascades, and Fc gamma R-mediated phagocytosis were also identified (Supplementary Table 6).

The functional annotation and enrichment among the 79 coexpressed DEGs in CON vs. DON and DON vs. GAD were investigated. GO annotation showed that most annotated BP ontology focused on immune cell migration such as myeloid leukocyte, leukocyte, neutrophil, mononuclear, and granulocyte. Furthermore, some DEGs were annotated as the external stimulus, inflammatory response, Notch binding, receptor binding, chemokine, chemokine receptor, IL-8 (CXCL8) receptor binding, and so on in the MF aspect (Supplementary Figure 1). After KEGG enrichment, DEGs including CXCL8, CCL5, and IL-15 were enriched in the chemokine and TNF signaling pathway and cytokine-cytokine receptor interaction pathway (Supplementary Table 7).

### 3.5. PPI Network Construction and Module Analysis

**Figure 6:**
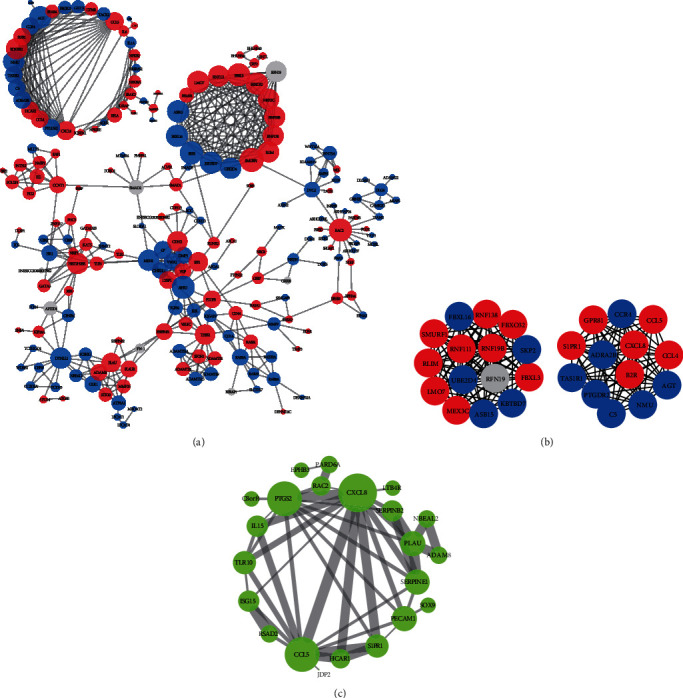
Protein-protein interaction (PPI) network construction of DEGs. (a) The PPI network of DEGs identified from CON vs. DON. (b) The top 30 highly connected DEGs with 19 edges of the PPI network and the top 2 significant modules extracted from the PPI network (MCODE scores are 15 and 13). (c) PPI network constructed from 79 coexpressed genes between CON vs. DON and DON vs. GAD. Red represents gene expression upregulated, and blue represents gene expression downregulated in (a) and (b). Node size indicates the number of genes interacted in (a) and (c). Line thickness indicates the strength of data support in (c). The interaction score was set to 0.9 in (a) and (b) and 0.4 for (c).

To further study the interaction among the DEGs, PPI networks were constructed in CON vs. DON. As shown in Figure 6(a), a network including 209 nodes and 572 relationship pairs was constructed, and genes including CXCL8, CCL4, IL-15, IL-6, IL1A, NFKB2, NFKB1A, and MAPK-related were highly interacted. The top 30 highly connected genes (hub genes) were searched out by cytoHubba, and the first 28 hub genes formed two independent networks, which were the top 2 significant modules including 15 nodes and 105 edges as well as 13 nodes and 78 edges extracted from Figure 6(a) by MCODE with scores of 15 and 13, respectively (Figure 6(b)). In order to investigate the interaction among genes involved in the alleviation of GA, the 79 DEGs coexpressed in both CON vs. DON and DON vs. GAD were used to construct the PPI network, and one network was obtained (Figure 6(c)). CXCL8 was the most hub gene interacted with a range of genes including CCL5, PTGS2, IL-15, HCAR1, RAC2, S1PR1, PECAM1, and LTB4R.

## 4. Discussion

Deoxynivalenol is the most prevalent mycotoxin, which is widespread and frequently exists in most human foods and animal feeds, resulting in intestinal diseases, systemic immunosuppression, and other diseases [31]. Pigs are more sensitive to DON than other kinds of animals. DON can lead to emesis, diarrhea, low nutrient absorption and growth, immune system disorders, and economical loss [32, 33]. It has been indicated that DON exposure can induce cytotoxicity, oxidative stress, apoptosis, and intestinal barrier dysfunction [34, 35]. It is also a ribotoxic mycotoxin to provoke oxidative stress and inflammatory responses [36]. Although the cytotoxic effects of DON have been well documented, few studies are conducted to alleviate its toxicity. Therefore, there is an urgent need to develop effective nutritional and protective strategies to alleviate the damage induced by DON and to improve the intestinal health of animals. GA has characters of anti-inflammation and liver protection, which is widely used to promote the growth and development in animals, but the protective effects of GA on alleviating DON-induced intestinal inflammation, apoptosis, and its active mechanism are rarely reported. Hence, this study provided the potential mechanism of GA for alleviating inflammation and apoptosis in DON-stimulated IPEC-J2 cells using molecular biochemistry techniques and RNA-seq analysis, which supported the application of GA as one kind of feed additive for alleviating DON cytotoxicity in animal feeding.

The recent study has revealed that DON can induce cellular oxidative stress and decrease the activities of antioxidant enzymes [37]. SOD and CAT are primary enzymes participating in repairing damage caused by oxidative stress. SOD can effectively eliminate O^2-^ to protect the body from the influence of superoxide anion, and CAT can decompose ROS produced in the metabolic process [38]. It was reported that DON could alter membrane integrity, cellular redox signaling, and antioxidant status of the cells [39]. MDA is the important product of membrane lipid peroxide to aggravate the damage of cell membrane, which can indirectly reflect the membrane integrity of cells [40]. This study showed that DON exposure significantly increased LDH release and MDA level and decreased the SOD and CAT activities, indicating DON cytotoxicity, in agreement with the previous report [11]; however, GA addition significantly increased the activities of these antioxidant enzymes and decreased the LDH release and MDA level, inferring that GA could alleviate the cytotoxicity and oxidative stress induced by DON.

In addition, the related proapoptotic and proinflammatory factors such as Bax, caspase 3, IL-6, IL-8, TNF-*α*, COX-2, and NF-*κ*B were significantly upregulated after DON stimulation in IPEC-J2 cells, which is in accordance with the previous study [11]. Proinflammatory cytokines such as IL-6, IL-8, and TNF-*α* are associated with the severity of inflammation and intestinal disease, which can directly affect intestinal epithelial cells. Excessive expression of them can result in damage to the epithelial barrier and secretion of chemokines such as COX-2 and NF-*κ*B, even causing epithelial cell apoptosis [41]. COX-2 is an immediate-early response gene, which is regarded as an inflammatory marker associated with a variety of inflammatory conditions and cancer [42]. NF-*κ*B plays a critical role in regulating inflammatory and immune responses as well as controlling cell proliferation and cell death [43]. Moreover, apoptosis is a genetically programmed procedure due to its central role in normal cell development and homeostasis at various physiological and pathological conditions [44, 45]. Bax, Bcl-2, and caspase 3 are the important mediators of cell apoptosis. Therefore, the upregulation of these genes in this study clearly indicated that DON exposure induced inflammation and apoptosis in IPEC-J2 cells. However, these proinflammatory and proapoptosis genes were downregulated by GA addition. In addition, the contents of IL-8, caspase 3, and NF-*κ*B in cell supernatant were increased after DON exposure, while GA could reduce their contents except for caspase 3. Furthermore, Annexin V-FITC/PI staining further confirmed that GA significantly decreased cell apoptosis induced by DON. The consistencies among the cytokine concentrations in cell supernatant, cytokine mRNA abundances, and protein expressions in this study prove the cytoprotective role of GA. Based on the above results, it was preliminary speculated that GA could act as a cytoprotective agent against DON-induced cytotoxicity, oxidative stress, inflammation, and apoptosis in IPEC-J2 cells.

To further characterize the potential mechanisms of GA for alleviating cell damage induced by DON, the RNA-seq was adopted to compare the differential gene expression profiles of different treatment groups in this study. The results showed that there were different numbers of DEGs during comparisons among 4 groups. GO and KEGG enrichment analyses of the DEGs were performed to analyze the functions of genes. It was found that the DEGs are primarily related to immune and inflammatory response. Cytokines and chemokines play critical parts in inflammation and immune response. Cytokines can directly promote or limit intestinal cell proliferation, permeability, and apoptosis, thereby destroying the intestinal epithelial barrier [46]. Chemokines are responsible for chemotactic cell migration and play important roles in the humoral and cellular immune responses to induce the production of corresponding inflammatory factors [47, 48]. Except for the proinflammatory factors, DON can also upregulate several chemokines. In the current study, the differentially expressed chemokines such as CCL4, CCL5, and CXCL8 were identified in the CON vs. DON. In general, CCL4 acts as a chemoattractant for a variety of immune cells [49], and CCL5 is a key proinflammatory chemokine [50], which can be produced by many kinds of cells including CD4^+^ T lymphocytes, epithelial cells, fibroblasts, and platelets [51]. CXCL8 can be induced by proinflammatory cytokines TNF-*α* [52]. It is evident that DON can activate the NF-*κ*B signaling pathway to trigger MAPK signaling pathways, which are essential for controlling inflammation by mediating the production of inflammatory factors [13]. A recent study demonstrated that inflammation occurred by activating the P38 MAPK and Erk1/2 pathway by comparing transcriptomes with and without DON treatment (2 *μ*g/mL for 2 h) in IPEC-J2 cells [53]. Although the same MAPK, TNF, NF-*κ*B signaling pathways, and cytokine-cytokine receptor interaction were enriched in this study, the number of shared DEGs in two studies was small when using the same DEG identification threshold. A total of 138 shared DEGs including CXCL8, IL1A, IL-6, FOS, and MAP3K5 were identified, in which only eight genes including FOS and IL1A showed an inconsistent expression state, mirroring the validity of results in both studies. The low proportion of overlapped DEGs suggests that there are some different pathways involved in inflammation induced by DON. Therefore, these findings highlight that TNF, MAPK, and NF-*κ*B signaling pathways and chemokines are involved in the inflammation and immune response of IPEC-J2 cells induced by DON.

GA has many functions of stimulating the antioxidant status and reinforcing the immune system function [54], which is also validated by RNA-seq analysis in this study. Most DEGs in CON vs. GA were annotated as the GO terms of cell development and the regulation of stimulus. The KEGG pathway enrichment analysis demonstrated that DEGs were significantly enriched into cell adhesion molecules and the immune system. Some pathways such as TNF and MAPK were insignificantly enriched in CON vs. GA. The similar pathways enriched in CON vs. DON were also enriched in CON vs. GAD, indicating that GA was involved in some of the same pathways to alleviate the damage from DON-induced IPEC-J2 cells. To uncover the potential pathway involved in the protection effect of GA, the DEGs in DON vs. GAD were investigated. Some of the 154 DEGs including CXCL8, IL-15, CCL5, and RAC2 in DON vs. GAD were enriched in chemokine, cytokine-cytokine receptor interaction, TNF, cAMP, and Jak-STAT signaling pathways, which were also included in the pathways enriched from CON vs. DON. It is demonstrated that chemokine, cytokine, and TNF signaling pathways may act as major contributors for the protection effect of GA.

This research showed that 79 DEGs coexpressed in both CON vs. DON and DON vs. GAD might be involved in the protection effect of GA. Among these DEGs, CXCL8, CCL5, and IL-15 interacted with each other in a high strength of data support were enriched to cytokine-cytokine receptor interaction and the TNF signaling pathway. This result further indicated that the TNF signaling pathway may participate the protection effect of GA. The 26 DEGs upregulated in the DON group and downregulated in the GAD group belonging to IL-15 and CCL5 enriched in the TNF signaling pathway and cytokine-cytokine receptor interaction. Furthermore, CCL5 and RAC2 are enriched in the chemokine signaling pathway, and RAC2 and HCAR1 are enriched in the cAMP signaling pathway. Particularly, CCL5 interacted with IL-15, ISG15, PTGS2, HCAR1, and CXCL8. CCL5 and IL-15 may act as key genes in the whole alleviation process of GA. IL-15 is a human neutrophil agonist known to induce RNA and de novo protein synthesis, phagocytosis, and apoptosis, which is also often regarded as a proinflammatory cytokine [55]. A previous study demonstrated that IL-15 can delay the apoptosis of neutrophil by recovering the expression of the antiapoptotic MCL1 protein via several kinases including Jak-2, p38, and ERK1/2 [56]. ISG15, a member of the family of ubiquitin-like proteins, is an important component of host responses to microbial infection [57]. For other DEGs, RAC2 and TXNIP are involved in oxidative stress, and LTB4R takes part in inflammatory and immune response. These results show that GA may alleviate the cell damage from DON via the TNF signaling pathway by downregulating IL-15 and CCL5 gene expressions, and other immunization-related and inflammation-related genes including ISG15, RAC2, TXNIP, and LTB4R also play important roles in the process.

## 5. Conclusion

**Figure 7:**
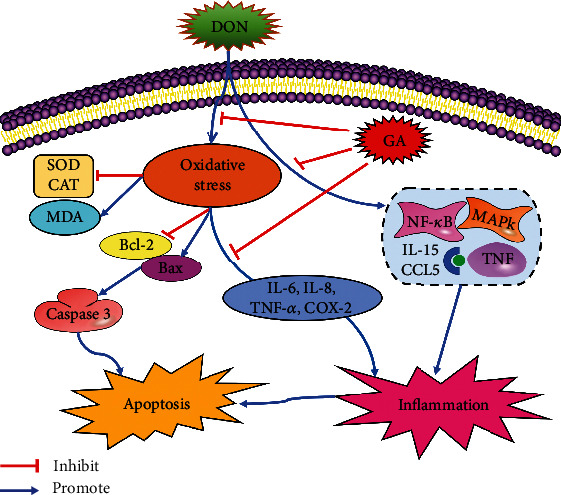
A proposed model of the protective mechanism of GA against the damage induced by mycotoxin exposure in intestinal epithelial cells of pigs.

DON exposure can induce oxidative stress, inflammation, and apoptosis in IPEC-J2 cells, which can be alleviated by GA addition. MAPK, TNF, and NF-*κ*B signaling pathways and some chemokines serve as major parts to participate in the inflammation and apoptosis induced by DON; however, GA shows alleviating effect via the TNF signaling pathway by downregulating IL-15, CCL5, and other immunization-related and inflammation-related gene expressions (Figure 7). This study provides a new insight into the protective mechanism of GA against the damage induced by mycotoxin exposure in intestinal epithelial cells of pigs, which also lays a theoretical foundation for the development and application of GA as a potential feed additive to alleviate DON-induced cytotoxicity for improving animal health and productions.

## Figures and Tables

**Table 1:** The summary of 63 coexpressed DEGs in CON vs. DON and DON vs. GAD.

Gene ID	Gene name	Full names of genes	CON vs. DON	CON vs. GA	CON vs. GAD	DON vs. GAD
ENSSSCG00000029311	MYPN	Myopalladin	Up	Down	—	Down
ENSSSCG00000006051	CTHRC1	Collagen triple helix repeat containing 1	Up	Down	Down	Down
ENSSSCG00000011972	FILIP1L	Filamin A interacting protein 1 like	Up	Down	—	Down
ENSSSCG00000008648	RSAD2	Radical S-adenosyl methionine domain containing 2	Up	Down	Down	Down
ENSSSCG00000035420	HES4	Hes family bHLH transcription factor 4	Up	Down	Down	Down
ENSSSCG00000029849	S1PR1	Sphingosine-1-phosphate receptor 1	Up	—	—	Down
ENSSSCG00000040359	GOLGA7B	Golgin A7 family member B	Up	—	Up	Down
ENSSSCG00000009789	HCAR1	Hydroxycarboxylic acid receptor 1	Up	—	—	Down
ENSSSCG00000025245	ARMC5	Armadillo repeat containing 5	Up	—	—	Down
ENSSSCG00000025134	FAM171B	Family with sequence similarity 171 member B	Up	—	Down	Down
ENSSSCG00000040575	ISG15	ISG15 ubiquitin-like modifier	Up	—	—	Down
ENSSSCG00000009051	IL-15	Interleukin 15	Up	—	—	Down
ENSSSCG00000017705	CCL5	C-C motif chemokine ligand 5	Up	—	—	Down
ENSSSCG00000021944	RAC2	Rac family small GTPase 2	Up	—	—	Down
ENSSSCG00000011195	GALNT15	Polypeptide N-acetylgalactosaminyltransferase 15	Up	—	Up	Down
ENSSSCG00000024158	ANO1	Anoctamin 1	Up	—	—	Down
ENSSSCG00000021027	PGBD5	PiggyBac transposable element-derived 5	Up	—	—	Down
ENSSSCG00000027911	LTB4R	Leukotriene B4 receptor	Up	—	—	Down
ENSSSCG00000009100	TNIP3	TNFAIP3-interacting protein 3	Up	—	Up	Down
ENSSSCG00000017277	PECAM1	Platelet and endothelial cell adhesion molecule 1	Up	—	Up	Down
ENSSSCG00000034870	—	—	Up	—	—	Down
ENSSSCG00000033093	RGS6	Regulator of G protein signaling 6	Up	—	—	Down
ENSSSCG00000026890	GALNT6	Polypeptide N-acetylgalactosaminyltransferase 6	Up	—	—	Down
ENSSSCG00000031262	TXNIP	Thioredoxin-interacting protein	Up	—	Up	Down
ENSSSCG00000003080	—	—	Up	—	—	Down
ENSSSCG00000001916	INSYN1	Inhibitory synaptic factor 1	Up	—	—	Down
ENSSSCG00000029438	SESN2	Sestrin 2	Down	Up	—	Up
ENSSSCG00000035895	JDP2	Jun dimerization protein 2	Down	Up	—	Up
ENSSSCG00000039761	MYCL	MYCL protooncogene, bHLH transcription factor	Down	Up	—	Up
ENSSSCG00000022649	SLC7A11	Solute carrier family 7 member 11	Down	Up	—	Up
ENSSSCG00000038521	CHAC1	ChaC glutathione-specific gamma-glutamylcyclotransferase 1	Down	Up	—	Up
ENSSSCG00000021232	SYNC	Syncoilin, intermediate filament protein	Down	Up	—	Up
ENSSSCG00000023749	—	—	Down	Up	—	Up
ENSSSCG00000008153	SLC9A2	Solute carrier family 9 member A2	Down	—	—	Up
ENSSSCG00000033059	CASKIN1	CASK-interacting protein 1	Down	—	Down	Up
ENSSSCG00000002778	ZDHHC1	Zinc finger DHHC-type containing 1	Down	—	—	Up
ENSSSCG00000016873	NIM1K	NIM1 serine/threonine protein kinase	Down	—	Down	Up
ENSSSCG00000011293	POMGNT2	Protein O-linked mannose N-acetylglucosaminyltransferase 2 (beta 1,4-)	Down	—	—	Up
ENSSSCG00000007486	CYP24A1	Cytochrome P450, family 24, subfamily A, polypeptide 1	Down	—	Down	Up
ENSSSCG00000029662	RASSF4	Ras association domain family member 4	Down	—	—	Up
ENSSSCG00000003848	LRP8	LDL receptor-related protein 8	Down	—	Down	Up
ENSSSCG00000025992	ENPP3	Ectonucleotide pyrophosphatase/phosphodiesterase 3	Down	—	—	Up
ENSSSCG00000027877	TLR10	Toll-like receptor 10	Down	—	—	Up
ENSSSCG00000016562	SSMEM1	Serine-rich single-pass membrane protein 1	Down	—	—	Up
ENSSSCG00000038026	RAB12	RAB12, member RAS oncogene family	Down	—	—	Up
ENSSSCG00000034863	PARD6A	Par-6 family cell polarity regulator alpha	Down	—	Down	Up
ENSSSCG00000011330	NBEAL2	Neurobeachin-like 2	Down	—	Down	Up
ENSSSCG00000011949	CEP97	Centrosomal protein 97	Down	—	—	Up
ENSSSCG00000014437	PPARGC1B	PPARG coactivator 1 beta	Down	—	—	Up
ENSSSCG00000035969	THRA	Thyroid hormone receptor alpha	Down	—	—	Up
ENSSSCG00000036172	SP9	Sp9 transcription factor	Down	—	Down	Up
ENSSSCG00000035876	ZNF599	Zinc finger protein 599	Down	—	—	Up
ENSSSCG00000024759	—	—	Down	—	—	Up
ENSSSCG00000011915	GRAMD1C	GRAM domain containing 1C	Down	—	—	Up
ENSSSCG00000026849	CCNO	Cyclin O	Down	—	—	Up
ENSSSCG00000003423	DRAXIN	Dorsal inhibitory axon guidance protein	Down	—	—	Up
ENSSSCG00000008227	ST3GAL5	ST3 beta-galactoside alpha-2,3-sialyltransferase 5	Down	—	—	Up
ENSSSCG00000031321	NR4A1	Nuclear receptor subfamily 4 group A member 1	Down	—	—	Up
ENSSSCG00000008413	GPR75	G protein-coupled receptor 75	Down	—	—	Up
ENSSSCG00000005673	IER5L	Immediate early response 5 like	Down	—	—	Up
ENSSSCG00000016420	INSIG1	Insulin-induced gene 1	Down	—	Down	Up
ENSSSCG00000037391	—	—	Down	—	—	Up
ENSSSCG00000007491	MC3R	Melanocortin 3 receptor	Down	—	—	Up

Note: CON: control group; DON: the single DON (0.5 *μ*g/mL) group; GA: the single GA (400 *μ*g/mL) group; GAD: 400 *μ*g/mL GA+0.5 *μ*g/mL DON group.

## Data Availability

The data used to support the findings of the present study are available from the corresponding authors upon request.

## Supplementary Materials

Supplementary Materials Supplementary Table S1: the detailed summary of sequencing data for each sample. Supplementary Table S2: the significant enriched KEGG pathways of the DEGs in CON vs. DON. Supplementary Table S3: the significant enriched KEGG pathways of the DEGs in CON vs. GA. Supplementary Table S4: the significant enriched KEGG pathways of the DEGs in CON vs. GAD. Supplementary Table S5: the significant enriched KEGG pathways of the DEGs in DON vs. GAD. Supplementary Table S6: the significant enriched KEGG pathways of the DEGs in CON vs. DON and DON vs. GAD. Supplementary Table S7: primer sequences of some genes for quantitative real-time PCR. Supplementary Figure S1: Gene Ontology (GO) annotation of DEGs in CON vs. DON and DON vs. GAD. The top 10 functional classified GO terms of DEGs annotated by subontology of GO analysis; BP: biological processes; MF: molecular function; CC: cellular components (CC).
