# Supplementary material for: Comparative Transcriptome Analysis Reveals the Protective Mechanism of Glycyrrhinic Acid for Deoxynivalenol-Induced Inflammation and Apoptosis in IPEC-J2 Cells
Source: Oxid Med Cell Longev. 2020 Oct 24;2020:5974157. doi: 10.1155/2020/5974157 (PMC7604610; doi:10.1155/2020/5974157)
Supplement: Supplementary Materials — Supplementary Table S1: the detailed summary of sequencing data for each sample. Supplementary Table S2: the significant enriched KEGG pathways of the DEGs in CON vs. DON. Supplementary Table S3: the significant enriched KEGG pathways of the DEGs in CON vs. GA. Supplementary Table S4: the significant enriched KEGG pathways of the DEGs in CON vs. GAD. Supplementary Table S5: the significant enriched KEGG pathways of the DEGs in DON vs. GAD. Supplementary Table S6: the significant enriched KEGG pathways of the DEGs in CON vs. DON and DON vs. GAD. Supplementary Table S7: primer sequences of some genes for quantitative real-time PCR. Supplementary Figure S1: Gene Ontology (GO) annotation of DEGs in CON vs. DON and DON vs. GAD. The top 10 functional classified GO terms of DEGs annotated by subontology of GO analysis; BP: biological processes; MF: molecular function; CC: cellular components (CC). [file 5974157.f1.docx]

**Supplementary Table S1.** The detailed summary of sequencing data for each sample

| **Sample** | **Raw reads** | **Raw Data (bp)** | **Q20 (%)** | **Q30 (%)** | **Clean Reads** | **Clean Reads %** | **Clean Data (bp)** | **Total Mapped** |
| --- | --- | --- | --- | --- | --- | --- | --- | --- |
| CON1 | 44,355,360 | 6,653,304,000 | 97.9 | 94.39 | 41,486,024 | 93.53 | 6,222,903,600 | 40,096,581 (96.65%) |
| CON2 | 43,872,658 | 6,580,898,700 | 98.01 | 94.65 | 41,011,722 | 93.47 | 6,151,758,300 | 39,758,034 (96.94%) |
| CON3 | 47,276,444 | 7,091,466,600 | 97.87 | 94.28 | 44,200,144 | 93.49 | 6,630,021,600 | 42,811,219(96.86%) |
| DON1 | 43,059,688 | 6,458,953,200 | 97.84 | 94.2 | 40,261,378 | 93.5 | 6,039,206,700 | 38,920,953 (96.67%) |
| DON2 | 46,610,024 | 6,991,503,600 | 98.03 | 94.67 | 43,634,072 | 93.61 | 6,545,110,800 | 42,240,202 (96.81%) |
| DON3 | 42,701,830 | 6,405,274,500 | 98.11 | 94.86 | 40,024,464 | 93.73 | 6,003,669,600 | 38,782,233 (96.90%) |
| GA1 | 48,394,126 | 7,259,118,900 | 98.06 | 94.75 | 45,392,396 | 93.79 | 6,808,859,400 | 43,130,338 (95.02%) |
| GA2 | 49,044,508 | 7,356,676,200 | 98 | 94.63 | 46,019,838 | 93.83 | 6,902,975,700 | 44,606,604 (96.93%) |
| GA3 | 49,471,102 | 7,420,665,300 | 98.06 | 94.74 | 46,197,432 | 93.38 | 6,929,614,800 | 44,793,482 (96.96%) |
| GAD1 | 40,626,224 | 6,093,933,600 | 97.96 | 94.5 | 38,073,658 | 93.71 | 5,711,048,700 | 36,818,160 (96.70%) |
| GAD2 | 43,235,144 | 6,485,271,600 | 97.78 | 94.07 | 40,438,914 | 93.53 | 6,065,837,100 | 39,059,611 (96.59%) |
| GAD3 | 41,128,124 | 6,169,218,600 | 97.93 | 94.42 | 38,476,614 | 93.55 | 5,771,492,100 | 37,194,228 (96.67%) |

Note: Q20 means sequence base error < 1%, Q30 means sequence base error < 0.1%.

**Supplementary Table S2.** The significant enriched KEGG pathways of the DEGs in CON vs. DON

| Pathway  ID | Pathway name | KEGG class | P-value | DEG  number | Total number |
| --- | --- | --- | --- | --- | --- |
| ssc04668 | TNF signaling pathway | Signal transduction | 7.74906E-07 | 20 | 69 |
| ssc04330 | Notch signaling pathway | Signal transduction | 5.92155E-05 | 11 | 33 |
| ssc04010 | MAPK signaling pathway | Signal transduction | 0.000108714 | 29 | 162 |
| ssc00100 | Steroid biosynthesis | Lipid metabolism | 0.000195367 | 7 | 16 |
| ssc05418 | Fluid shear stress and atherosclerosis | Cardiovascular diseases | 0.000220126 | 19 | 91 |
| ssc05202 | Transcriptional misregulation in cancer | Cancers: Overview | 0.000242345 | 20 | 99 |
| ssc04014 | Ras signaling pathway | Signal transduction | 0.000328743 | 24 | 132 |
| ssc04658 | Th1 and Th2 cell differentiation | Immune system | 0.001480557 | 13 | 60 |
| ssc04064 | NF-kappa B signaling pathway | Signal transduction | 0.00173679 | 13 | 61 |
| ssc04662 | B cell receptor signaling pathway | Immune system | 0.002014901 | 10 | 41 |
| ssc04933 | AGE-RAGE signaling pathway in diabetic complications | Endocrine and metabolic diseases | 0.003627559 | 13 | 66 |
| ssc05222 | Small cell lung cancer | Cancers: Specific types | 0.004765704 | 11 | 53 |
| ssc01522 | Endocrine resistance | Drug resistance: Antineoplastic | 0.005158796 | 12 | 61 |
| ssc05200 | Pathways in cancer | Cancers: Overview | 0.00630639 | 40 | 311 |
| ssc04380 | Osteoclast differentiation | Development | 0.006922658 | 13 | 71 |
| ssc04620 | Toll-like receptor signaling pathway | Immune system | 0.012368712 | 11 | 60 |
| ssc04392 | Hippo signaling pathway - multiple species | Signal transduction | 0.020781329 | 4 | 13 |
| ssc01040 | Biosynthesis of unsaturated fatty acids | Lipid metabolism | 0.020781329 | 4 | 13 |
| ssc04068 | FoxO signaling pathway | Signal transduction | 0.024393629 | 13 | 83 |
| ssc05167 | Kaposi's sarcoma-associated herpesvirus infection | Infectious diseases: Viral | 0.025519874 | 15 | 101 |
| ssc05164 | Influenza A | Infectious diseases: Viral | 0.025519874 | 15 | 101 |
| ssc00900 | Terpenoid backbone biosynthesis | Metabolism of terpenoids and polyketides | 0.027156418 | 4 | 14 |
| ssc05134 | Legionellosis | Infectious diseases: Bacterial | 0.027290761 | 7 | 35 |
| ssc00512 | Mucin type O-glycan biosynthesis | Glycan biosynthesis and metabolism | 0.029769671 | 5 | 21 |
| ssc04917 | Prolactin signaling pathway | Endocrine system | 0.032115637 | 8 | 44 |
| ssc04670 | Leukocyte transendothelial migration | Immune system | 0.032662102 | 11 | 69 |
| ssc05030 | Cocaine addiction | Substance dependence | 0.034230396 | 6 | 29 |
| ssc04657 | IL-17 signaling pathway | Immune system | 0.035508601 | 9 | 53 |
| ssc04024 | cAMP signaling pathway | Signal transduction | 0.039065035 | 17 | 125 |
| ssc05221 | Acute myeloid leukemia | Cancers: Specific types | 0.04068182 | 8 | 46 |
| ssc04640 | Hematopoietic cell lineage | Immune system | 0.041624727 | 10 | 63 |
| ssc05033 | Nicotine addiction | Substance dependence | 0.042712328 | 5 | 23 |
| ssc04218 | Cellular senescence | Cell growth and death | 0.043763369 | 13 | 90 |
| ssc05132 | Salmonella infection | Infectious diseases: Bacterial | 0.045484786 | 8 | 47 |
| ssc05133 | Pertussis | Infectious diseases: Bacterial | 0.045484786 | 8 | 47 |

**Supplementary Table S3.** The significant enriched KEGG pathways of the DEGs in CON vs. GA

| Pathway  ID | Pathway name | KEGG class | P-value | DEG  number | Total number |
| --- | --- | --- | --- | --- | --- |
| ssc04514 | Cell adhesion molecules (CAMs) | Signaling molecules and interaction | 4.31414E-05 | 8 | 80 |
| ssc04670 | Leukocyte transendothelial migration | Immune system | 0.000120316 | 7 | 69 |
| ssc05160 | Hepatitis C | Infectious diseases: Viral | 0.00062817 | 7 | 90 |
| ssc04610 | Complement and coagulation cascades | Immune system | 0.00149236 | 5 | 52 |
| ssc00533 | Glycosaminoglycan biosynthesis - keratan sulfate | Glycan biosynthesis and metabolism | 0.01087347 | 2 | 10 |
| ssc00830 | Retinol metabolism | Metabolism of cofactors and vitamins | 0.013602825 | 3 | 31 |
| ssc04640 | Hematopoietic cell lineage | Immune system | 0.019093395 | 4 | 63 |
| ssc05202 | Transcriptional misregulation in cancer | Cancers: Overview | 0.022455284 | 5 | 99 |
| ssc04664 | Fc epsilon RI signaling pathway | Immune system | 0.026906358 | 3 | 40 |
| ssc04115 | p53 signaling pathway | Cell growth and death | 0.026906358 | 3 | 40 |
| ssc04530 | Tight junction | Cellular community - eukaryotes | 0.027093649 | 5 | 104 |
| ssc04964 | Proximal tubule bicarbonate reclamation | Excretory system | 0.03051253 | 2 | 17 |
| ssc05217 | Basal cell carcinoma | Cancers: Specific types | 0.032453073 | 3 | 43 |
| ssc04151 | PI3K-Akt signaling pathway | Signal transduction | 0.039728991 | 7 | 195 |
| ssc04360 | Axon guidance | Development | 0.040587531 | 5 | 116 |
| ssc03320 | PPAR signaling pathway | Endocrine system | 0.045187527 | 3 | 49 |
| ssc04068 | FoxO signaling pathway | Signal transduction | 0.046074626 | 4 | 83 |
| ssc04072 | Phospholipase D signaling pathway | Signal transduction | 0.047791426 | 4 | 84 |

**Supplementary Table S4.** The significant enriched KEGG pathways of the DEGs in CON vs. GAD

| Pathway  ID | Pathway name | KEGG class | P-value | DEG  number | Total number |
| --- | --- | --- | --- | --- | --- |
| ssc05202 | Transcriptional misregulation in cancer | Cancers: Overview | 2.55126E-05 | 20 | 99 |
| ssc04668 | TNF signaling pathway | Signal transduction | 2.77945E-05 | 16 | 69 |
| ssc04064 | NF-kappa B signaling pathway | Signal transduction | 9.88141E-05 | 14 | 61 |
| ssc00100 | Steroid biosynthesis | Lipid metabolism | 0.000639858 | 6 | 16 |
| ssc04010 | MAPK signaling pathway | Signal transduction | 0.001581185 | 23 | 162 |
| ssc04330 | Notch signaling pathway | Signal transduction | 0.002133103 | 8 | 33 |
| ssc04390 | Hippo signaling pathway | Signal transduction | 0.002620505 | 15 | 92 |
| ssc04014 | Ras signaling pathway | Signal transduction | 0.003346682 | 19 | 132 |
| ssc05219 | Bladder cancer | Cancers: Specific types | 0.004082948 | 6 | 22 |
| ssc04514 | Cell adhesion molecules (CAMs) | Signaling molecules and interaction | 0.005076849 | 13 | 80 |
| ssc05200 | Pathways in cancer | Cancers: Overview | 0.00688489 | 35 | 311 |
| ssc04068 | FoxO signaling pathway | Signal transduction | 0.006968209 | 13 | 83 |
| ssc04670 | Leukocyte transendothelial migration | Immune system | 0.011032952 | 11 | 69 |
| ssc04658 | Th1 and Th2 cell differentiation | Immune system | 0.011105727 | 10 | 60 |
| ssc04392 | Hippo signaling pathway - multiple species | Signal transduction | 0.011972685 | 4 | 13 |
| ssc04657 | IL-17 signaling pathway | Immune system | 0.013885697 | 9 | 53 |
| ssc04640 | Hematopoietic cell lineage | Immune system | 0.015468759 | 10 | 63 |
| ssc05206 | MicroRNAs in cancer | Cancers: Overview | 0.020460886 | 12 | 85 |
| ssc05224 | Breast cancer | Cancers: Specific types | 0.02063393 | 13 | 95 |
| ssc05164 | Influenza A | Infectious diseases: Viral | 0.032408328 | 13 | 101 |
| ssc01522 | Endocrine resistance | Drug resistance: Antineoplastic | 0.032415349 | 9 | 61 |
| ssc05418 | Fluid shear stress and atherosclerosis | Cardiovascular diseases | 0.033028236 | 12 | 91 |
| ssc04380 | Osteoclast differentiation | Development | 0.033192683 | 10 | 71 |
| ssc05134 | Legionellosis | Infectious diseases: Bacterial | 0.039630571 | 6 | 35 |
| ssc04060 | Cytokine-cytokine receptor interaction | Signaling molecules and interaction | 0.040139332 | 17 | 147 |
| ssc05165 | Human papillomavirus infection | Infectious diseases: Viral | 0.045594853 | 21 | 194 |
| ssc04550 | Signaling pathways regulating pluripotency of stem cells | Cellular community - eukaryotes | 0.048549375 | 11 | 86 |

**Supplementary Table S5.** The significant enriched KEGG pathways of the DEGs in DON vs. GAD

| Pathway  ID | Pathway name | KEGG class | P-value | DEG  number | Total number |
| --- | --- | --- | --- | --- | --- |
| ssc04062 | Chemokine signaling pathway | Immune system | 0.002497511 | 5 | 108 |
| ssc04071 | Sphingolipid signaling pathway | Signal transduction | 0.004140903 | 4 | 75 |
| ssc04664 | Fc epsilon RI signaling pathway | Immune system | 0.005073299 | 3 | 40 |
| ssc04060 | Cytokine-cytokine receptor interaction | Signaling molecules and interaction | 0.009205517 | 5 | 147 |
| ssc04610 | Complement and coagulation cascades | Immune system | 0.010530736 | 3 | 52 |
| ssc04666 | Fc gamma R-mediated phagocytosis | Immune system | 0.014831892 | 3 | 59 |
| ssc05323 | Rheumatoid arthritis | Immune diseases | 0.014831892 | 3 | 59 |
| ssc04024 | cAMP signaling pathway | Signal transduction | 0.023837162 | 4 | 125 |
| ssc04668 | TNF signaling pathway | Signal transduction | 0.02246974 | 3 | 69 |
| ssc04670 | Leukocyte transendothelial migration | Immune system | 0.02246974 | 3 | 69 |
| ssc00480 | Glutathione metabolism | Metabolism of other amino acids | 0.020009963 | 2 | 25 |
| ssc04216 | Ferroptosis | Cell growth and death | 0.028211798 | 2 | 30 |
| ssc05144 | Malaria | Infectious diseases: Parasitic | 0.035578411 | 2 | 34 |
| ssc05206 | MicroRNAs in cancer | Cancers: Overview | 0.038384334 | 3 | 85 |
| ssc04630 | Jak-STAT signaling pathway | Signal transduction | 0.049297796 | 3 | 94 |

**Supplementary Table S6.** The significant enriched KEGG pathways of the DEGs in CON vs. DON and DON vs. GAD

| Pathway ID | Pathway name | KEGG class | P-value | DEG  number | Total number |
| --- | --- | --- | --- | --- | --- |
| ssc04062 | Chemokine signaling pathway | Immune system | 0.001272228 | 4 | 108 |
| ssc05323 | Rheumatoid arthritis | Immune diseases | 0.002217861 | 3 | 59 |
| ssc04668 | TNF signaling pathway | Signal transduction | 0.003467466 | 3 | 69 |
| ssc04060 | Cytokine-cytokine receptor interaction | Signaling molecules and interaction | 0.003920422 | 4 | 147 |
| ssc05144 | Malaria | Infectious diseases: Parasitic | 0.009885966 | 2 | 34 |
| ssc05164 | Influenza A | Infectious diseases: Viral | 0.010009271 | 3 | 101 |
| ssc04024 | cAMP signaling pathway | Signal transduction | 0.017749803 | 3 | 125 |
| ssc04610 | Complement and coagulation cascades | Immune system | 0.022228673 | 2 | 52 |
| ssc04620 | Toll-like receptor signaling pathway | Immune system | 0.0290228 | 2 | 60 |
| ssc04064 | NF-kappa B signaling pathway | Signal transduction | 0.029923897 | 2 | 61 |
| ssc00604 | Glycosphingolipid biosynthesis - ganglio series | Glycan biosynthesis and metabolism | 0.035092909 | 1 | 8 |
| ssc04670 | Leukocyte transendothelial migration | Immune system | 0.037524529 | 2 | 69 |
| ssc04080 | Neuroactive ligand-receptor interaction | Signaling molecules and interaction | 0.039830678 | 3 | 171 |
| ssc05142 | Chagas disease (American trypanosomiasis) | Infectious diseases: Parasitic | 0.042612346 | 2 | 74 |
| ssc04071 | Sphingolipid signaling pathway | Signal transduction | 0.043659584 | 2 | 75 |

**Supplementary** **Table S7.** Primer sequences of some genes for quantitative real-time PCR

| **Gene** | **Accession number** | **Primer sequence (5’-3’)** |
| --- | --- | --- |
| GAPDH | XM-004387206 | F: ATGACCACAGTCCATGCCATC |
|  |  | R: CCTGCTTCACCACCTTCTTG |
| IL-6 | NM_214399 | F: GCTCTCTGTGAGGCTGCAGTTC |
|  |  | R: AAGGTGTGGAATGCGTATTTATGC |
| IL-8 | NM_213867 | F: GACCCCAAGGAAAAGTGGGT |
|  |  | R: TGACCAGCACAGGAATGAGG |
| TNF-α | NM_214022 | F: TTCCAGCTGGCCCCTTGAGC |
|  |  | R: GAGGGCATTGGCATACCCAC |
| COX-2 | NM_214321 | F: AG AAGASTGGACCAGCTTTC |
|  |  | R: AAAGCGGAGGTGTTCAGGAG |
| NF-κB | NC_006509021 | F: CTCGCACAAGGAGACATGAA |
|  |  | R: ACTCAGCCGGAAGGCATTAT |
| Bax | XM-003355975.1 | F: ATGATCGCAGCCGTGGACACG |
|  |  | R: AASTAGATGGTCACCGTCTGC |
| Bcl-2 | XM-003122573.2 | F: AGAGCCGTTTCGTCCCTTTC |
|  |  | R: GCACGTTTCCTAGASTGCAT |
| Caspase-3 | NM-214131.1 | F: TTGGACTGTGGGATTGAGACG |
|  |  | R: CGCTGCACAAAGTGACTGGA |

Note: GAPDH, Glyceraldehyde-3-phosphate dehydrogenase; IL-6, Interleukin 6; IL-8, Interleukin 8; TNF-α, Tumor necrosis factor α; COX-2, Cyclooxygenase-2; NF-κB, Nuclear factor kappa B; Bcl-2, B-cell lymphoma-2.


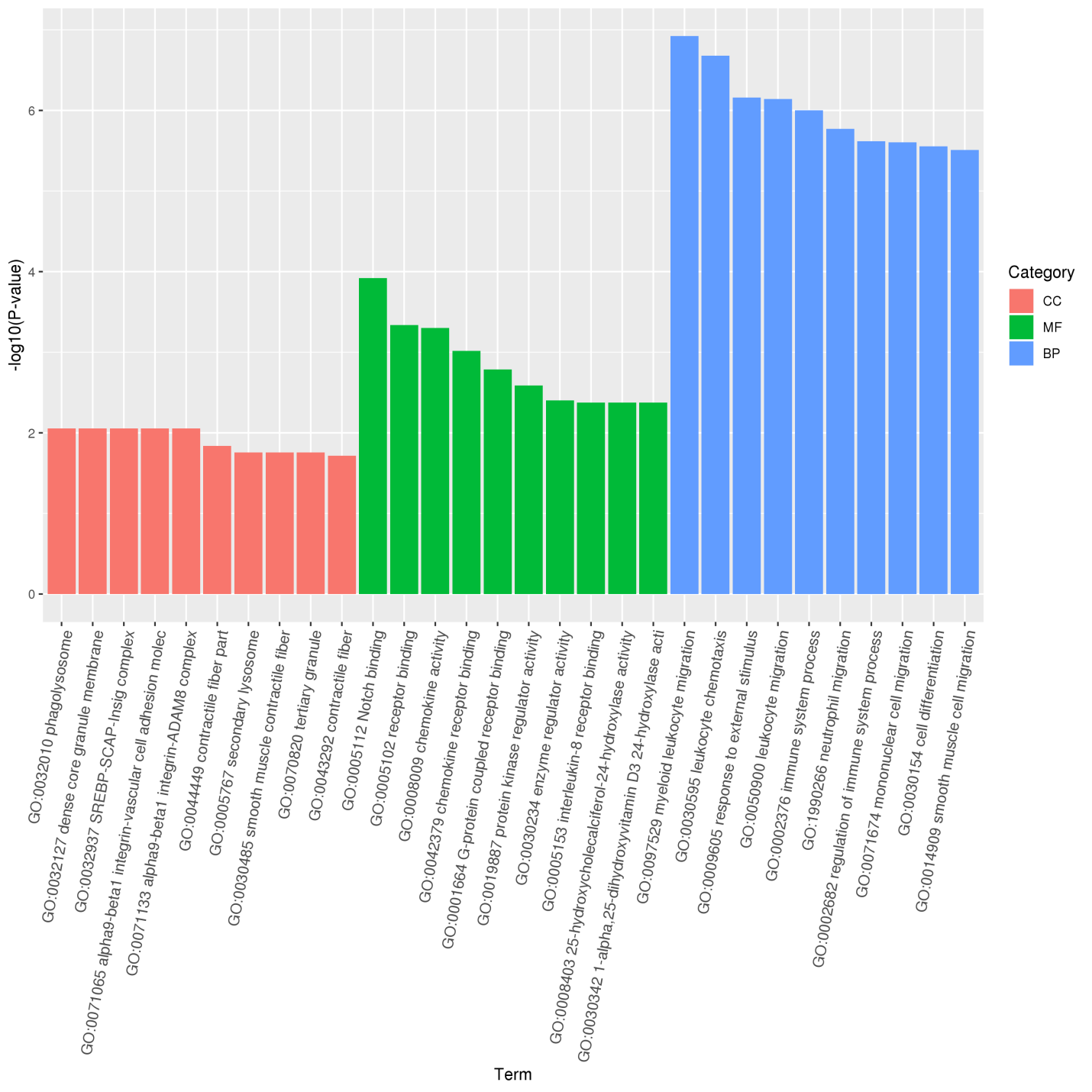


**Supplementary** **Figure S1.** Gene Ontology (GO) annotation of DEGs in CON vs. DON and DON vs. GAD. The top 10 functional classified GO terms of DEGs annotated by sub-ontology of GO analysis; BP: biological processes, MF: molecular function, CC: cellular components (CC).
